# Silymarin administration after cerebral ischemia improves survival of obese mice by increasing cortical BDNF and IGF1 levels

**DOI:** 10.3389/fnagi.2024.1484946

**Published:** 2024-11-18

**Authors:** Yesica María Rodríguez-Cortés, Ricardo Jair Ramírez-Carreto, Julia Isabel Rodríguez-Barrena, Marelly Salazar-Castro, Anahí Chavarría

**Affiliations:** ^1^Facultad de Medicina, Unidad de Medicina Experimental “Ruy Pérez Tamayo”, Universidad Nacional Autónoma de México, Mexico City, Mexico; ^2^Programa de Doctorado en Ciencias Biomédicas, Univesidad Nacional Autónoma de México, Mexico City, Mexico

**Keywords:** BDNF, cerebral ischemia, neuroinflammation, obesity, silymarin, systemic inflammation

## Abstract

**Background:**

Obesity is associated with a systemic inflammatory state that contributes to neuroinflammation and increases the risk of stroke at an early age. Stroke is the third leading cause of death worldwide and the leading cause of permanent disability. This work aimed to assess whether obesity-induced neuroinflammation can be a prognostic stroke factor that can be improved with oral administration of silymarin, an anti-inflammatory and neuroprotective drug.

**Methods:**

Male C57/Bl6 mice were used to establish an obesity model through a high-fat diet (HFD) for 12 weeks. Cerebral ischemia was performed with photothrombosis in the left motor cortex at the end of the diet. Following the induction of ischemia, silymarin (100 mg/kg) was administered orally for 14 days. Levels of pro-inflammatory (IL1β, TNFα, and MCP1) and anti-inflammatory markers (IL4, IL10), neurotrophic factors (IGF1, BDNF), and CX3CL1 were assessed in the cortex and striatum using ELISA.

**Results:**

Mice on the HFD gained significantly more weight than control subjects and exhibited altered glucose metabolism, which was improved after silymarin treatment. The survival rate was significantly lower in HFD mice (52.2%) compared to control mice (86%). Silymarin treatment improved survival in both ischemic groups (non-diet control: 95.7%, HFD: 78.3%). Silymarin raised cortical TNFα, IL4, IL10, IGF1, BDNF, and CX3CL1 levels in the HFD group with stroke, while the striatum did not present relevant differences.

**Conclusion:**

Our findings suggest that silymarin improves glucose metabolism, possibly impacting post-stroke survival in obese mice. The increased levels of neurotrophic factors BDNF and IGF1, along with microglial regulatory factor CX3CL1, may contribute to the improved survival observed. These results indicate that silymarin could be a potential therapeutic option for managing neuroinflammation and enhancing post-stroke outcomes in obese individuals.

## 1 Introduction

Obesity is a global health problem, representing the sixth cause of death worldwide (Hruby and Hu, 2015). According to the World Health Organization (WHO), in 2016, more than 650 million adults worldwide were classified as obese (World Health Organization, 2021), a number that continues to rise. By 2035, the World Obesity Federation estimates nearly 2 billion individuals, including adults, children, and adolescents, will be obese, with the associated healthcare costs surpassing those of the COVID-19 pandemic in 2020 (Federation, 2023).

Obesity is characterized by adipocyte hypertrophy and hyperplasia, which results in a chronic low-grade inflammatory state (Longo et al., 2019). This inflammatory environment arises from the chronic release of pro-inflammatory cytokines such as TNFα, IL6, and ILβ by visceral adipose tissue, triggering inflammatory pathways such as NFκB or JNK that, in the long term, lead to complications such as insulin resistance (Singer and Lumeng, 2017). Moreover, this inflammation compromises the permeability of the blood–brain barrier (BBB) by decreasing the expression of claudins and occludins, enabling inflammatory molecules to infiltrate the central nervous system (CNS) (Van Dyken and Lacoste, 2018). In the CNS, microglia and astrocytes are activated by these circulating inflammatory agents—TNFα, free fatty acids, and LPS—via Toll-like receptors, further perpetuating neuroinflammation in brain regions such as the hippocampus, cortex, brainstem, and amygdala (Amor et al., 2014; Van Dyken and Lacoste, 2018; Saieva and Taglialatela, 2022).

Obesity is also a well-established independent risk factor for cerebral ischemia, with the World Stroke Organization (WSO) listing high body mass index as one of the leading causes of stroke (Feigin et al., 2022). In individuals aged 15 to 49 with stroke, obesity has been linked to an increased risk of early-onset ischemic stroke, likely mediated by hypertension and diabetes mellitus (Mitchell et al., 2015). Animal studies further illustrate this risk, showing that obese mice present a larger infarct area and more significant neuronal loss than lean mice, as obesity amplifies post-ischemic inflammatory response in animals, impacting their recovery (Haley and Lawrence, 2016). In obese patients, elevated levels of inflammatory markers such as C-reactive protein (CRP), IL6, TNFα, MCP1, and ICAM-1 have been associated with higher risks of cerebral infarction (Haley and Lawrence, 2016).

Cerebral infarction, which includes both ischemic and hemorrhagic stroke, is the second cause of death and permanent disability worldwide, with ischemic stroke accounting for over 80% of cases (Musuka et al., 2015; Saini et al., 2021). This condition progresses through acute, subacute, and chronic stages, leaving permanent neurological deficits due to primary and secondary neuronal loss (Musuka et al., 2015). Animal models, particularly the photothrombotic model of cerebral ischemia, are instrumental in studying these pathological processes. The photothrombotic stroke model induces cerebral ischemia via photosensitive dye and targeted light exposure, creating precise, reproducible cortical infarctions with minimal invasiveness. This model is especially valuable for studying post-ischemic pathways like cell death and inflammation, as it causes localized ischemic damage within the cortex (Clarkson et al., 2013; Labat-gest and Tomasi, 2013). Furthermore, because the model ensures high survival rates, it enables long-term investigations of sensorimotor deficits, making it a robust tool for assessing neuroprotective interventions (Uzdensky, 2018).

In parallel, high-fat diet (HFD)-induced obesity models are widely used to replicate human metabolic conditions in animals. In these models, rodents are fed diets with increased fat content to mimic obesity-related metabolic changes such as insulin resistance, hyperlipidemia, and chronic inflammation (Buettner et al., 2007). The HFD model is particularly relevant for studying the interplay between obesity and stroke, as it mirrors the inflammatory and metabolic dysfunctions seen in obese patients, which worsen ischemic outcomes (Maysami et al., 2015; Grisotto et al., 2021). By combining the photothrombotic stroke and HFD-induced obesity models, researchers can explore how obesity exacerbates stroke pathology and how therapeutic interventions might mitigate the synergistic effects of obesity and cerebral ischemia.

One promising avenue for neuroprotection in cerebral ischemia is silymarin, a standardized extract derived from the seeds and fruits of *Silybum marianum*. 80% of silymarin is composed of four flavonolignan isomers (silybin, isosilybin, silydianin, and silychristin), while 20% contain an unidentified chemical fraction of polyphenolic compounds (Wellington and Jarvis, 2001). Flavonolignans comprise 70% silybin, the main active compound responsible for most of its pharmacological effects such as antioxidant activity, anxiolytic, anti-inflammatory, and neuroprotective (Wellington and Jarvis, 2001; Ranjan and Gautam, 2023). These attributes make silymarin an attractive candidate for treating cerebral ischemia, where inflammation, oxidative stress, and cell death are prominent factors.

Preclinical studies have shown that when administered before ischemia, silymarin mitigated tissue damage by modulating inflammatory pathways and reducing the activation of pro-inflammatory mediators, like NF-kB and STAT-1 pathways, improving neurological outcomes (Hou et al., 2010). In the middle cerebral artery occlusion (MCAO) model, silymarin pretreatment demonstrated a dose-dependent antioxidant effect, reducing apoptosis and enhancing functional recovery (Raza et al., 2011). This is significant because oxidative stress plays a critical role in the pathophysiology of cerebral ischemia since it contributes to cell death (Allen and Bayraktutan, 2009).

Given that over 60% of stroke patients are ineligible for recanalization therapy, they are more likely to experience long-term cognitive impairment (Ma et al., 2020; Cerasuolo et al., 2022). Thus, cerebral ischemia remains a leading cause of disability, imposing substantial burdens on patients, their families, and healthcare systems. This study aims to evaluate whether silymarin can improve survival and motor outcomes in obese mice subjected to permanent cerebral ischemia through the photothrombotic model, offering insights into potential neuroprotective strategies for stroke recovery.

## 2 Methods

### 2.1 Animals

All experiments were performed in 12-weeks-old male C57BL/6 J mice (25–30 g), which were maintained with food and water *ad libitum* under a 12:12 light–dark cycle.

All experimental procedures followed the Mexican Law of Animal Protection for the use and care of laboratory animals (Norma Official Mexicana NOM-062-ZOO-1999) and the Guidelines for the Use of Animals in Neuroscience Research of the Society of Neuroscience. This project was approved by the Comisiones de Investigación y Ética (Approval FM/DI/119/2019) and the Comité Interno para el Cuidado y Uso de Animales de Laboratorio (CICUAL 035-CIC-2019) of Facultad de Medicina, Universidad Nacional Autónoma de México. We reduced the number of mice used and their suffering or pain as much as possible.

### 2.2 Obesity model and silymarin treatment

Obesity was induced in mice by administering a high-fat diet (HFD) for 3 months, consisting of 38% fat, 38% carbohydrates, and 16% protein, while control mice were fed a standard laboratory diet (ND) with 6% fat, 49% carbohydrates, and 24% protein, as described by Buettner et al. (2007)(Buettner et al., 2007).

Following the induction of obesity, both ND and HFD groups underwent photothrombosis and were randomly assigned to receive either silymarin (S0292 Sigma-Aldrich, MO, United States; 100 mg/kg of body weight administered orally for 14 consecutive days) or vegetable oil as a vehicle (Muley et al., 2013; Ramirez-Carreto et al., 2023). Intragastric administration in mice was performed daily at 8:00 h with an oral gavage (curved needle, 20G x1 ½ in). Each mouse was trained for 3 days before the administration scheme to recognize the curved gavage needle and for the restraining technique. During the protocol performance, mice were gently restrained, immobilizing the head and avoiding signs of distress. The total administration volume was calculated as 8 mL/Kg, and necropsies were performed to discard misadministration issues for mice who died during the photothrombosis protocol.

Animals were euthanized 15 days after photothrombosis.

### 2.3 Metabolic follow-up of the animals

During the induction of the obesity period, weight gain was determined weekly, and biochemical analyses were done to determine the metabolic state.

ND and HFD mice treated with silymarin or vehicle were assigned to an intraperitoneal glucose or insulin tolerance curve.

For the glucose and insulin tolerance tests, animals fasted for 12 h. Each animal received an intraperitoneal injection with glucose (2 g/kg) or insulin (rapid recombinant human insulin, 1 U/kg). Blood glucose concentration was measured using a standard glucometer (Freestyle Optium Neo, Abbott) at 0, 15, 30, 60, and 120 min post-injection. After the procedure, the mice were left in their boxes with food and water on demand.

### 2.4 Cerebral ischemia

Mice were anesthetized with 2% isoflurane and maintained with 1% in an oxygen/air mixture using an anesthetic gas mask in a stereotaxic setting to induce the ischemic event. Three minutes before illumination, 0.3 mL of rose bengal solution (198,250 Sigma-Aldrich, MO, United States) was injected intraperitoneally in isotonic saline at 10 mg/mL concentration. The skull was exposed through a midline skin incision and illuminated with a fiberoptic beam from a 4.5 mm aperture cold-light source centered 1.5 mm to the left of the bregma for 8 min (Lopez-Valdes et al., 2014). The scalp was sutured after illumination, and the mice were allowed to awaken from anesthesia.

The animals were observed for 15 days post-ischemia, during which the behavioral motor evaluation test was performed. Trimethoprim-sulfamethoxazole at 2 mg/mL was dispensed orally for 8 days in drinking water to avoid infections. No analgesics were administered to prevent modifying the inflammatory response pathways.

### 2.5 Cylinder test

The cylinder test was performed before ischemia and at weeks one and two post-ischemia.

The animal was recorded while exploring a cylinder 10 cm in diameter and 20 cm high for 10 min to observe the differential use of its limbs from various angles and correlate it with the ischemic event. The videos were evaluated for the analysis by observing the times the animal sustained its paw ipsi- or contralateral to the lesion on the cylinder wall.

A double-blind system was used to avoid bias in evaluating behavioral trials. This method ensured that observers were unaware of the distribution of the experimental groups, thus minimizing potential influences on data collection and analysis. It also allowed behavioral tests to be standardized between observers and ensured consistent and reliable results in the analysis of motor exploration.

### 2.6 Cytokine titration

Pro- (IL1β, and TNF*α*) and anti-inflammatory cytokines (IL4 and IL10), monocyte chemoattractant protein (MCP1), CX3CL1, insulin-like growth factor 1 (IGF1) and brain-derived neurotrophic factor (BDNF) were determined by ELISA in cortex and striatum from the ipsilateral region of the lesion (DuoSet Mouse TNF-α DY410, DuoSet Mouse IL-1β/IL-1F2 DY401, DuoSet Mouse IL-4 DY4045, DuoSet Mouse IL-6 DY406, DuoSet Mouse IL-10 Dy417, DuoSet Mouse CX3CL1/Fractalkine DY472, DuoSet Mouse/Rat IGF-1/IGF-I DY791, and DuoSet Human/Mouse BDNF Dy248 ELISA kits, R&D Systems, Minneapolis, MN, United States). Mice were euthanized with intraperitoneal sodium pentobarbital (150 mg/kg). Brain regions were dissected in ice and transferred to microcentrifuge tubes containing 500 μL of lysis buffer (20 mM Tris, 0.25 M sucrose, 2 mM EDTA, 10 mM EGTA, 1% Triton X-100) and a protease inhibitor cocktail. Tissues were homogenized and centrifuged at 15,000 rpm for 30 min at 4°C, and then the supernatant was recovered and kept at −70°C until processing. The assays were performed following the manufacturer’s instructions. Optical density readings were made at 450 nm and corrected with 570 nm wavelength. All assays were performed by duplicate.

### 2.7 Statistical analysis

Analysis was done using GraphPad Prism 10.0. Results from all experiments were subjected to normality (D’Agostino-Pearson test) and homoscedasticity (Brown-Forsythe test) tests to determine parametric data. Groups were compared using the Student T test, one-way or two-way ANOVA, and Tukey’s post-hoc test. Mortality was analyzed using the log-rank Mantel-Cox test. Data are presented as mean ± sem. *p* ≤ 0.05 was considered significant.

## 3 Results

### 3.1 High-fat diet-induced weight gain and impaired glucose metabolism

**Figure 1:**
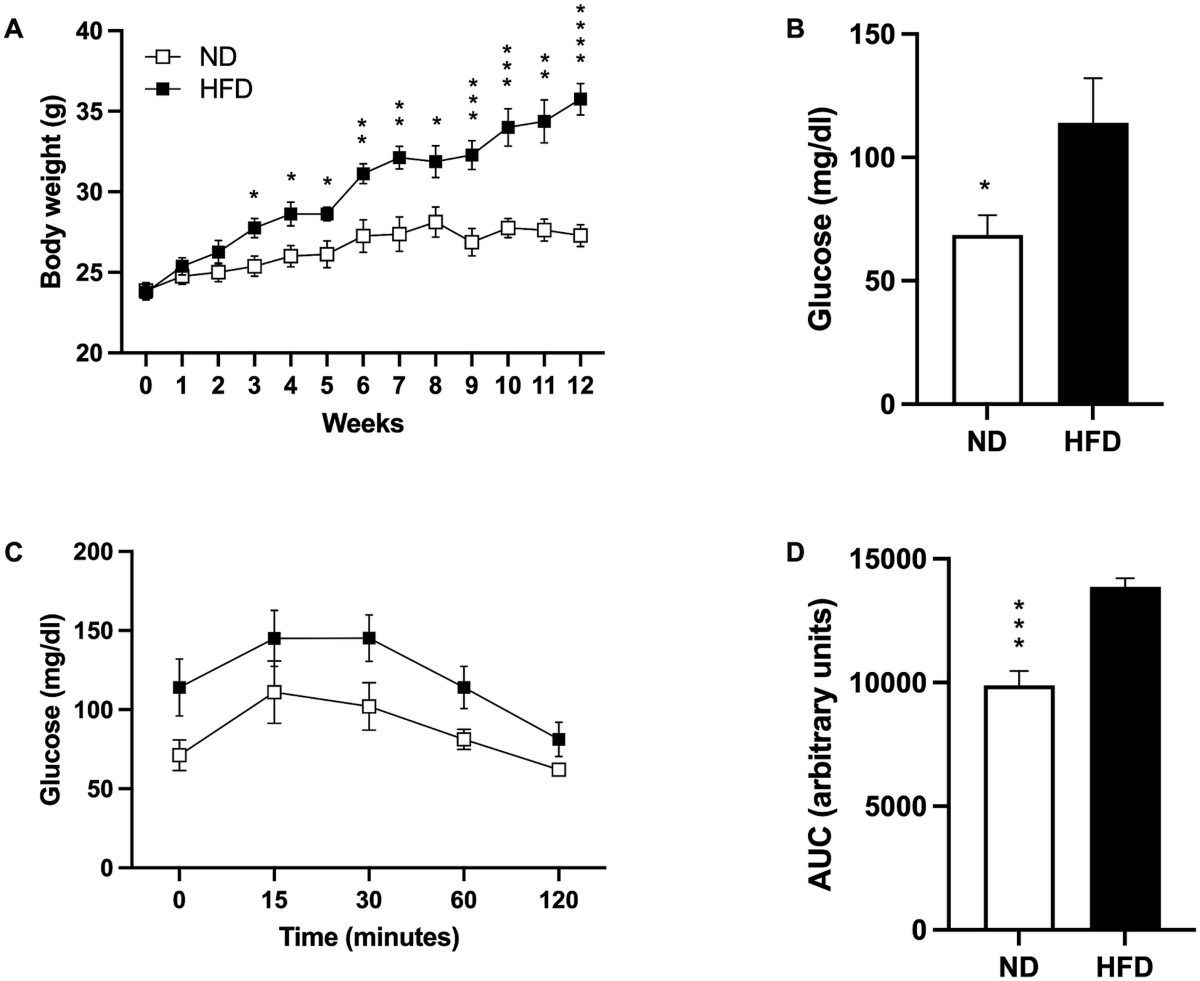
Induction of the obesity model. (A) Weight gain in mice fed a normal diet (ND; *n* = 8) and high-fat diet (HFD; *n* = 8). Data presented are mean ± SEM and were analyzed with two-way ANOVA with a Tukey *post hoc* test. (B) Basal glucose levels of ND (*n* = 5) and HFD (*n* = 5). Data presented are mean ± SEM and were analyzed with a T-test. (C) Intraperitoneal glucose tolerance curve of ND (*n* = 4) and HFD (*n* = 4). Data presented are mean ± SEM and were analyzed with two-way ANOVA with a Tukey *post hoc* test. (D) Area under the curve (AUC) for the intraperitoneal glucose tolerance test of ND and HFD. Data presented are mean ± SEM and were analyzed with a *t*-test. *p ≤ 0.05, **p ≤ 0.01, p*** ≤ 0.001, and p**** < 0.0001.

At the beginning of HFD model induction, both groups started with similar body weights (ND 23.88 gr ± 0.4795; HFD 23.75 gr ± 0.4532). Significant weight differences appeared by week 3 (*p* = 0. 0153), becoming highly significant at the end of the diet (*p* < 0.0001; Figure 1A).

As expected, basal fasting glucose levels were higher in the HFD group (*p* = 0.0419; Figure 1B), and while the glucose tolerance test showed a slightly higher post-load level at 15, 30, 60, and 120 min in the HFD group, no statistical significances were found (Figure 1C). However, analysis of the area under the curve (AUC) indicated that the HFD-fed mice presented more AUC than the ND group (*p* = 0.001; Figure 1D).

### 3.2 Silymarin effectively restored glucose metabolism

**Figure 2:**
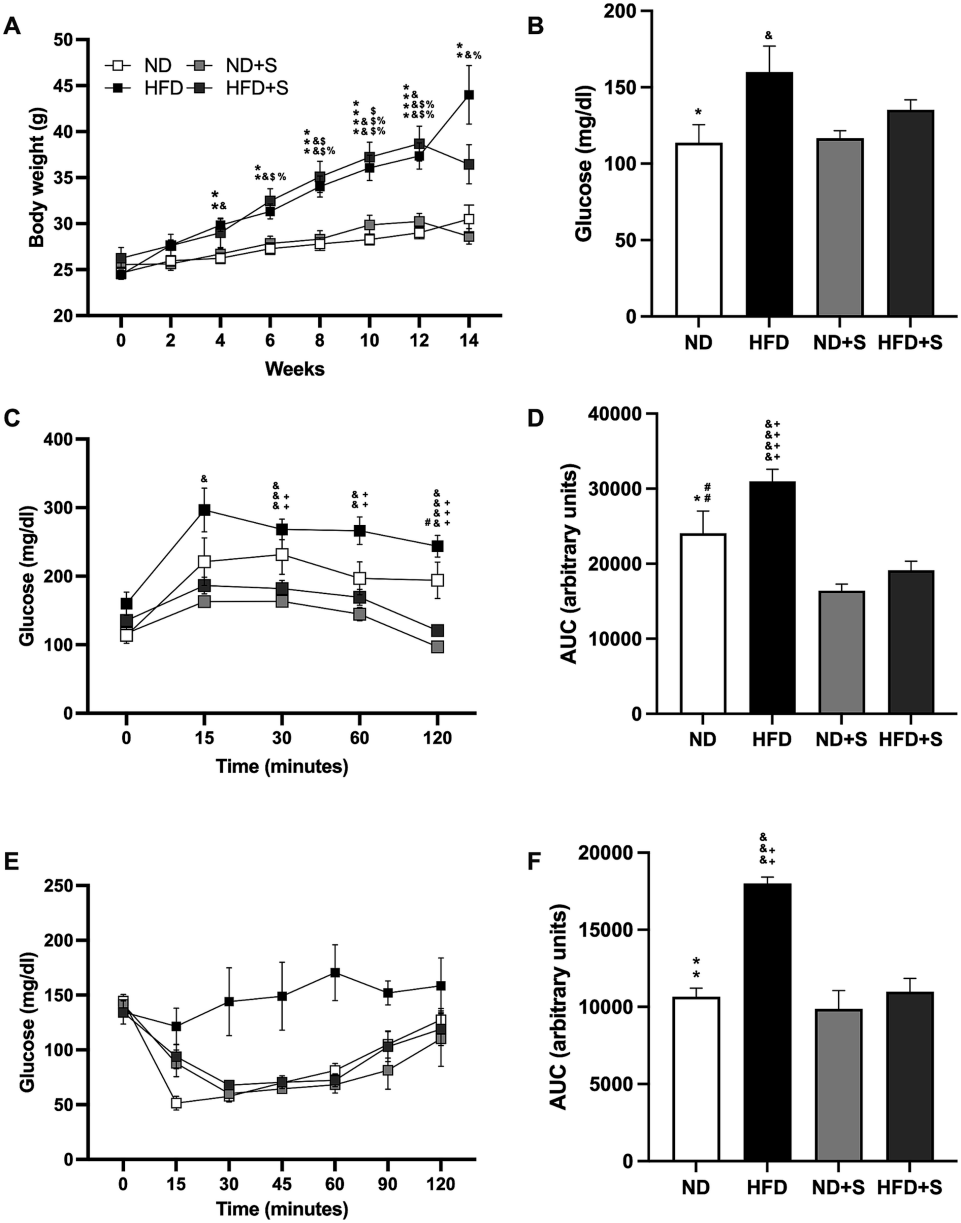
Effect of silymarin in the obesity model. (A) Weight gain in mice fed a normal diet (ND; *n* = 22), high-fat diet (HFD; *n* = 22), ND with silymarin treatment (ND + S = 14), and HFD with silymarin treatment (HFD + S = 15). Mice received 14 days of 100 mg/kg of silymarin oral treatment after the obesity induction diet. Data presented are mean ± SEM and were analyzed with two-way ANOVA with a Tukey *post hoc* test. (B) Basal glucose levels of ND (*n* = 7), HFD (*n* = 7), ND + S (*n* = 12), and HFD + S (*n* = 12) groups. Data presented are mean ± SEM and were analyzed with one-way ANOVA with a Tukey *post hoc* test. (C) Intraperitoneal glucose tolerance curve of ND (*n* = 7), HFD (*n* = 7), ND + S (*n* = 12), and HFD + S (*n* = 12) groups. Data presented are mean ± SEM and were analyzed with two-way ANOVA with a Tukey *post hoc* test. (D) Area under the curve (AUC) for the intraperitoneal glucose tolerance test of ND, HFD, ND + S, and HFD + S. Data presented are mean ± SEM and were analyzed with one-way ANOVA with a Tukey *post hoc* test. (E) Insulin tolerance test of the ND (*n* = 5), HFD (*n* = 4), ND + S (*n* = 4), and HFD + S (*n* = 5) groups. Data presented are mean ± SEM and were analyzed with two-way ANOVA with a Tukey *post hoc* test. (F) Area under the curve (AUC) for the insulin tolerance test of the ND, HFD, ND + S, and HFD + S groups. Data presented are mean ± SEM and were analyzed with one-way ANOVA with a Tukey *post hoc* test. ND compared to HFD group: *p ≤ 0.05, **p ≤ 0.01, p*** ≤ 0.001, and p**** < 0.0001. ND compared to HFD + S group: $*p* ≤ 0.05, $$p ≤ 0.01, $$$*p* ≤ 0.001. HFD compared to ND + S group: &*p* ≤ 0.05, &&*p* ≤ 0.01, &&&*p* ≤ 0.001, &&&&*p* < 0.0001. ND + S compared to HFD + S group: %*p* ≤ 0.05, %%*p* ≤ 0.01. ND compared to ND + S group: #*p* ≤ 0.05, ##*p* ≤ 0.01. HFD compared to HFD + S group: ++*p* ≤ 0.01, +++*p* ≤ 0.001, ++++*p* < 0.0001.

Initially, all groups had similar body weights (ND 24.64 gr ± 0.6191; HFD 24.45 gr ± 0.5085; ND + S 25.54 gr ± 0.6851; HFD 26.23 gr ± 1.172). By week 4, weight differences between ND and HFD groups became significant (ND vs. HFD *p* = 0.0011; ND + S vs. HFD *p* = 0.0106). By week 6, all HFD groups were significantly heavier than the ND groups (ND vs. HFD *p* = 0.0017; ND vs. HFD + S *p* = 0.0127; ND + S vs. HFD *p* = 0.0218; ND + S vs. HFD + S *p* = 0.0357), with differences more pronounced by week 12 (ND vs. HFD *p* < 0.0001; ND vs. HFD + S p = 0.0011; ND + S vs. HFD *p* = 0.0009; ND + S vs. HFD + S *p* = 0.0042; Figure 2A). Silymarin had no effect in the ND or HFD groups, and weight differences between the ND groups and the HFD mice were preserved, although HFD + S mice were not significantly different from the ND group at the end of the diet (ND vs. HFD *p* = 0.0249; ND + S vs. HFD *p* = 0.0265; ND + S vs. HFD + S *p* = 0.015; Figure 2A).

The glucose tolerance test revealed that HFD mice had higher glucose levels at 15, 30, 60, and 120 min compared to ND + S (*p* = 0.0211, *p* = 0.0006, *p* = 0.002, p < 0.0001, respectively; Figure 2C), and significantly higher glucose levels at 30, 60, and 120 min compared to HFD + S (*p* = 0.027, *p* = 0.0085, *p* = 0.0003, respectively; Figure 2C). Silymarin treatment also reduced glucose levels at 120 min in ND + S mice compared to ND controls (*p* = 0.0311; Figure 2C). AUC analysis confirmed that HFD had a larger area than ND (*p* = 0.0467), ND + S (p < 0.0001), and HFD + S groups (p < 0.0001) and that ND + S mice had a smaller AUC than ND controls (*p* = 0.0091; Figure 2D).

Although the insulin tolerance test showed no significant differences in post-load glucose levels at 15, 30, 60, and 120 min among the groups (Figure 2E), HFD groups exhibited a significantly larger AUC than ND (*p* = 0.0016), ND + S (*p* = 0.0009), and HFD + S groups (*p* = 0.0024; Figure 2F).

Interestingly, silymarin improved glucose and insulin tolerance tests in the HFD group (Figures 2C–F) and enhanced glucose metabolism in the ND group (Figures 2C,D).

### 3.3 Silymarin improved survival after stroke in obese mice

**Figure 3:**
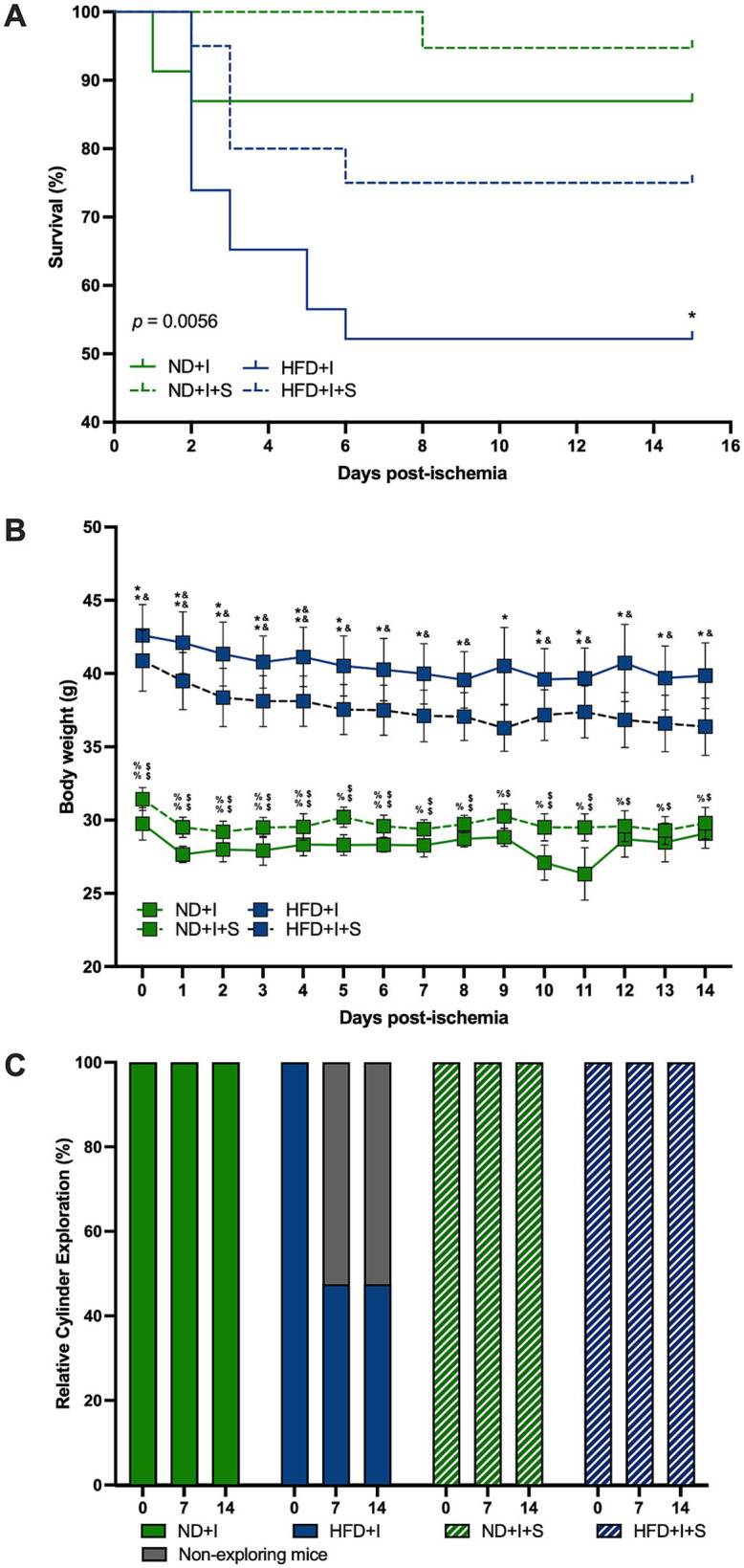
Silymarin effects on the clinical outcome after cerebral ischemia. (A) Survival curves of mice submitted to cerebral ischemia fed a normal diet (ND + I; *n* = 23), high-fat diet (HFD + I; *n* = 23), ND + I with silymarin treatment (ND + I + S; *n* = 19), and HFD + I with silymarin treatment (HFD + I + S; *n* = 20). Data were analyzed using the log-rank Mantel-Cox test. *p ≤ 0.05, ND + I compared to HFD + I group. (B) Body weight loss in ND + I (*n* = 4), HFD + I (*n* = 5), ND + I + S (*n* = 7), and HFD + I with silymarin treatment (HFD + I + S = 8). Mice received 14 days of 100 mg/kg of silymarin oral treatment after the photothrombosis. Data presented are mean ± SEM and were analyzed with two-way ANOVA with a Tukey *post hoc* test. (C) Percentage of ND + I (*n* = 8), HFD + I (*N* = 7), ND + I + S (*n* = 5), and HFD + I + S (*n* = 8) mice exploring the cylinder. Data were analyzed with Chi-square. ND + I compared to HFD + I group: *p ≤ 0.05, **p ≤ 0.01. ND + I compared to HFD + I + S group: $*p* ≤ 0.05, $$*p* ≤ 0.01. HFD + I compared to ND + I + S group: &*p* ≤ 0.05, &&*p* ≤ 0.01. ND + I + S compared to HFD + I + S group: %*p* ≤ 0.05, %%*p* ≤ 0.01.

The survival curves differed significantly among experimental groups (*p* = 0.0056; Figure 3A). Only 53% of HFD mice survived cerebral ischemia, compared to 87% in the ND group (*p* = 0.0195). Silymarin treatment improved survival in both HFD and ND mice (75 and 95%, respectively; Figure 3A).

Notably, ND mice subjected to photothrombosis did not experience significant weight loss (Figure 3B). However, HFD mice with cerebral ischemia lost weight on days 6, 7, and 8 compared to day 1 (1 vs. 6 *p* = 0.0284, 1 vs. 7 *p* = 0.0076, 1 vs. 8 *p* = 0.0079), and on day five compared to day four after photothrombosis (*p* = 0.0177). Silymarin treatment in the ND + I induced weight loss on days 1 and 6 compared to the beginning body weight (0 vs. 1 *p* = 0.0013, 0 vs. 6 *p* = 0.0201) and on day five compared to day 6 (*p* = 0.0441). Silymarin-treated HFD + I mice lost weight on days 2, 6, 7, and 8 compared to baseline (0 vs. 2 *p* = 0.0146, 0 vs. 6 *p* = 0.0288, 0 vs. 7 *p* = 0.0123, 0 vs. 8 *p* = 0.0167).

Silymarin-treated ND and HFD groups with cerebral ischemia showed no significant differences in body weight over the 14 days of treatment compared to their respective controls with photothrombosis (Figure 3B). The main significant differences were between the two diets, ND and HFD, independently of silymarin treatment (Figure 3B).

### 3.4 Cerebral ischemia impaired the motor behavior of animals, and silymarin improved it

HFD mice exhibited severe motor deficits after cerebral ischemia, as measured by the cylinder test. Some mice did not even perform the test. Due to this situation and mortality, we present the data of mice who performed the exploration during the cylinder test, with the HFD group significantly different (*p* < 0.0001); silymarin administration reversed this deficit (Figure 3C), consistent with improved survival.

### 3.5 Silymarin modulated the postischemia-induced TNFα levels in the cerebral cortex of HFD mice

**Figure 4:**
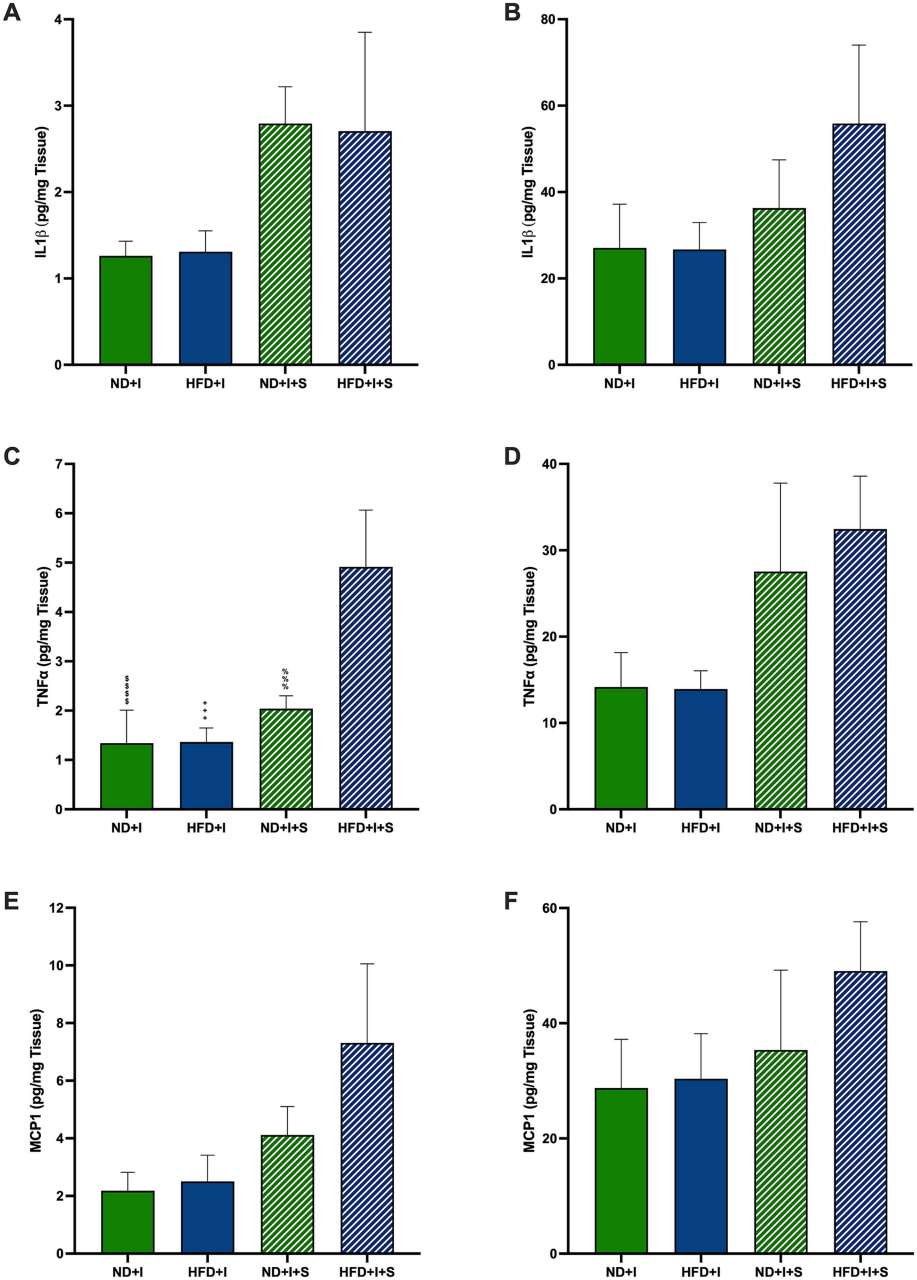
Effect of silymarin on cortical and striatal levels of inflammatory cytokines and MCP1. Cortical (A,C,E) and striatal (B,D,F) of IL1β (A,B), TNFα (C,D), and MCP1 (E and F) were determined in mice submitted to cerebral ischemia fed a normal diet (ND + I; *n* = 4–8), high-fat diet (HFD + I; *n* = 4–7), ND + I with silymarin treatment (ND + I + S; *n* = 3), and HFD + I with silymarin treatment (HFD + I + S; *n* = 3–4). Data presented are mean ± SEM and were analyzed with one-way ANOVA with a Tukey *post hoc* test. ND + I compared to HFD + I + S group: $$$$*p* < 0.0001. HFD + I compared to HFD + I + S group: ++++*p* < 0.0001. ND + I + S compared to HFD + I + S group: %%%*p* ≤ 0.001.

Several cytokines and neurotrophic factors were determined to evaluate further how silymarin could improve survival in HFD mice after cerebral ischemia. After 14 days of silymarin, IL1β tended to rise in the cerebral cortex of ND mice compared to ND + I and HFD + I + S (*p* = 0.0781 and *p* = 0.0736, respectively; Figure 4A), while no changes were observed in the striatum (Figure 4B). Silymarin treatment in HFD submitted to photothrombosis showed a slight increase in both cerebral regions (Figures 4A,B). TNFα levels were significantly elevated in the cerebral cortex of silymarin-treated HFD mice compared to all other groups (*p* < 0.0001 compared to ND + I and HFD + I, and *p* = 0.0007 compared to ND + I + S; Figure 4C), although no changes were observed in the striatum (Figure 4D). MCP1 levels showed no significant differences across experimental groups (Figures 4E,F).

### 3.6 Silymarin treatment after cerebral ischemia increased cortical modulatory molecules in HFD mice

**Figure 5:**
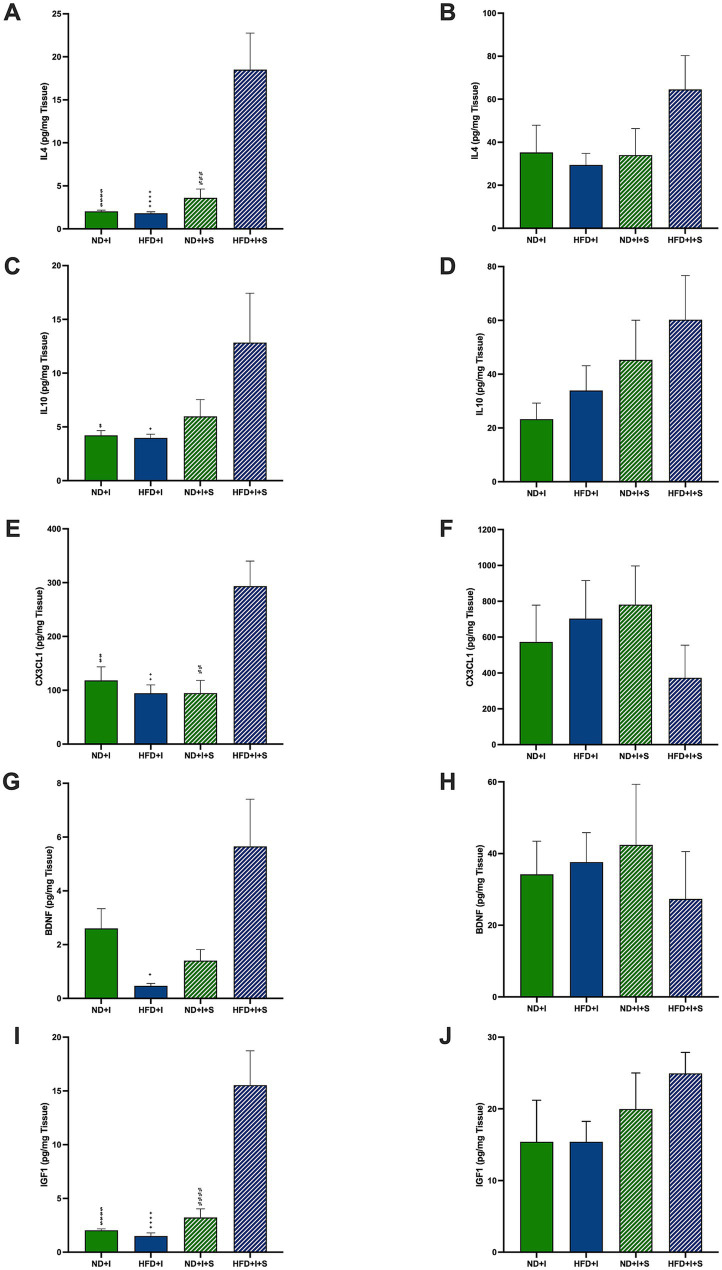
Effect of silymarin on cortical and striatal levels of modulatory molecules. Cortical (A,C,E,G,I) and striatal (B,D,F,H,J) of IL4 (A,B), IL10 (C,D), and MCP1 (E,F) were determined in mice submitted to cerebral ischemia fed a normal diet (ND + I; *n* = 3–9), high-fat diet (HFD + I; *n* = 4–5), ND + I with silymarin treatment (ND + I + S; *n* = 3–4), and HFD + I with silymarin treatment (HFD + I + S; *n* = 3–5). Data presented are mean ± SEM and were analyzed with one-way ANOVA with a Tukey *post hoc* test. ND + I compared to HFD + I + S group: $*p* ≤ 0.05, $$p ≤ 0.01, $$$$*p* < 0.0001. HFD + I compared to HFD + I + S group: +*p* ≤ 0.05, ++*p* ≤ 0.01, ++++*p* < 0.0001. ND + I + S compared to HFD + I + S group: %%*p* ≤ 0.01, %%%*p* ≤ 0.001, %%%%*p* < 0.0001.

Among the modulatory molecules that could be involved in the post-ischemia outcome of HFD mice are IL4 and IL10. Silymarin-treated HFD mice presented cortical IL4 significantly higher than the other experimental groups (*p* < 0.0001; Figure 5A). In the striatum, no significant differences were observed (Figure 5B). Also, HFD submitted to photothrombosis treated with silymarin increased cortical IL10 compared to the ND + I and HFD + I control groups (*p* = 0.0432 and *p* = 0.0291, respectively; Figure 5C); no changes were detected in the striatum (Figure 5D).

Additionally, cortical CX3CL1, fractalkine, a key microglial regulatory chemokine, was significantly increased in the HFD + I + S mice (*p* = 0.0091 compared to ND + I, *p* = 0.0022 compared to HFD + I, *p* = 0.0068 compared to ND + I + S; Figure 5E), suggesting its role in reducing excitotoxic damage and improving stroke outcomes. In the striatum, silymarin had no effect (Figure 5F).

Neurotrophic factors are relevant neuroprotective molecules; herein, we determined BDNF and IGF1 in the cortex and striatum 14 days after photothrombosis. BDNF was higher in HFD + I + S mice than in the HFD + I group (*p* = 0.0108) and tended to be higher compared to the ND + I + S group (*p* = 0.0589; Figure 5G). Additionally, IGF1 was significantly elevated after treatment of HFD mice than in the other experimental groups (p < 0.0001, Figure 5I), further indicating its neuroprotective potential. No significant changes were detected in the striatum (Figures 5H,J).

## 4 Discussion

This article investigated the impact of obesity on stroke prognosis while exploring the potential therapeutic effects of silymarin, known for its anti-inflammatory and neuroprotective properties (Moghaddam et al., 2020; Habotta et al., 2023; Haddadi et al., 2023; Li et al., 2023). Male C57/Bl6 mice were placed on a high-fat diet (HFD) for 12 weeks to induce obesity. Notably, HFD mice showed signs of weight gain as early as 3 weeks, which persisted throughout the study, emphasizing the early and sustained impact of an HFD on weight (Figure 1A; Moreno-Fernandez et al., 2018; Nagy and Einwallner, 2018; Rodriguez-Correa et al., 2020). Fasting glucose and intraperitoneal glucose tolerance tests in the HFD mice indicated potential metabolic syndrome development (Figures 1B,C), a well-known risk factor for cerebral vascular events (Ak et al., 2022; Zuo et al., 2023).

Considering that obesity is a primary risk factor for type 2 diabetes (Bays et al., 2023), the risk of an ischemic stroke increases in the younger population with diabetes (Emerging Risk Sarwar et al., 2010; Chen et al., 2016; O’Donnell et al., 2016). Moreover, in humans, the sole higher body mass index is an established risk factor for developing ischemic stroke in both men and women (Kurth et al., 2002; Kroll et al., 2016). Our data highlighted obesity as a substantial risk factor for stroke, as demonstrated by increased mortality in obese mice subjected to induced cerebral ischemia (Figure 3A). Additionally, obesity severity correlated with impaired motor function, as evidenced by poorer performance on the cylinder test, a deficit reversed by silymarin treatment (Figure 3C). Silymarin treatment also improved post-ischemia survival in all photothrombotic-exposed mice (Figure 3A). Interestingly, unlike other models of cerebral ischemia, stroke-induced mice had no critical weight loss, and silymarin had no effect on this parameter (Figure 3B).

We measured several pro- and anti-inflammatory cytokines, CX3CL1, and neurotrophic factors at 14 days post-photothrombosis to explore silymarin’s neuroprotective mechanisms. We found no significant differences between the ischemic HFD and ND mice in either the cortex or striatum (Figures 4, 5), suggesting that surviving HFD mice responded similarly to ND mice regarding molecular profiles. Notably, silymarin’s effects were restricted to the cortex of HFD-treated mice. The penumbra, an area with hypoperfusion and potential for neuron rescue, represents a therapeutic target in post-ischemia interventions (Walther et al., 2023). In our model, the motor cortex was the primary infarct zone, while the striatum was the penumbra. Therefore, silymarin’s beneficial effect on the cortex likely reflects its role in tissue rescue and regeneration. This is supported by the observed increases in the anti-inflammatory cytokines IL-4 and IL-10 in the cortex (Figures 5A,C), both of which are known to regulate neuroinflammation and are predominantly produced by activated glial cells and T lymphocytes (Schwartz and Baruch, 2014; Kawabori and Yenari, 2015).

Fractalkine (CX3CL1) is another key molecule that may modulate neuroinflammation (Poniatowski et al., 2017). Produced primarily by neurons and endothelial cells in the cortex, hippocampus, and other structures (Nishiyori et al., 1998; Tarozzo et al., 2003), CX3CL1 interacts with its receptor, CX3CR1, on microglia and macrophages to regulate microglial activations (Harrison et al., 1998; Zujovic and Taupin, 2003). Additionally, CX3CL1 is relevant in controlling glutamate excitotoxicity and TNFα secretion by microglia (Zujovic et al., 2000; Limatola et al., 2005). Our results show a significant cortical increase in CX3CL1 in obese animals treated with silymarin (Figure 5E), suggesting that CX3CL1 might regulate excitotoxic damage by modulating glutamate release after ischemia, thus promoting neuronal survival. Elevated plasma CX3CL1 has been associated with better human stroke outcomes (Donohue et al., 2012).

At the subacute stage, 14 days post-ischemia, silymarin did not affect MCP1 levels (Figure 4E), indicating a more nuanced timeline of its therapeutic influence. However, TNFα was the only pro-inflammatory cytokine elevated in HFD treated with silymarin after the ischemic event, which was accompanied by elevated levels of the anti-inflammatory cytokines IL4 and IL10, suggesting that TNFα may play a dual role, maintaining an inflammatory environment while also modulating synaptic strength and excitatory transmission in the cortex (Beattie et al., 2002; Pickering et al., 2005; Stellwagen and Malenka, 2006; Bras et al., 2020; Wang et al., 2023). In ischemic stroke models, TNFα has a pro-inflammatory effect in the acute phase but protective effects in later stages, as seen in hippocampal tissue (Bruce et al., 1996). This may explain the motor improvements observed in the cylinder test, where silymarin restored exploratory behavior in HFD mice 14 days after stroke (Figure 3C).

Importantly, silymarin’s influence on neurotrophic factors, as evidenced by increased BDNF levels, aligns with existing literature highlighting its anti-inflammatory and antioxidant effects in several neurological-related models, including cerebral ischemia, translating into improved motor function and BDNF content recovery in animals (Song et al., 2016; Thakare et al., 2017; Yon et al., 2019; Moghaddam et al., 2020; Shokouhi et al., 2020; Ramirez-Carreto et al., 2023).

Although direct evidence linking silymarin to increased IGF-1 levels is lacking, its potential role in enhancing insulin sensitivity opens avenues for exploring the intricate signaling cascades shared by insulin and IGF-1. This hypothesis is probable since our data show that obese mice treated with silymarin improved metabolically, reflected in glucose and insulin tolerance tests (Figures 2C–F). IGF-1, produced by microglia, astrocytes, and neurons, likely contributes to neuroprotection by modulating glutamate excitotoxicity in cerebral ischemia models (Hayes et al., 2021; Ge et al., 2022; Hayes et al., 2023), promoting functional outcomes.

In summary, our study demonstrates that silymarin improves survival and motor outcomes in obese mice following cerebral ischemia, with potential clinical implications for obese stroke patients. Elevated anti-inflammatory cytokines (IL-4 and IL-10) and neurotrophic factors (BDNF, IGF-1) in silymarin-treated mice suggest that silymarin may offer neuroprotective benefits by mitigating obesity-related neuroinflammation. Given that obesity is a significant risk factor for stroke, silymarin could be a promising adjunct therapy for improving post-stroke outcomes, particularly in patients not eligible for recanalization therapies like tissue plasminogen activator due to the short therapeutic window, leaving patients with limited treatment options (Kang et al., 2021). Additionally, its ability to improve glucose metabolism and insulin tolerance further highlights silymarin’s potential in managing comorbid conditions, such as diabetes, which is prevalent among obese stroke patients (Ruze et al., 2023). Future clinical trials should investigate whether these findings translate to improved outcomes in human stroke patients.

## Data Availability

The raw data supporting the conclusions of this article will be made available by the authors, without undue reservation.
